# Proton Beam Therapy without Fiducial Markers Using Four-Dimensional CT Planning for Large Hepatocellular Carcinomas

**DOI:** 10.3390/cancers10030071

**Published:** 2018-03-14

**Authors:** Satoshi Shibata, Shigeyuki Takamatsu, Kazutaka Yamamoto, Miu Mizuhata, Sayuri Bou, Yoshitaka Sato, Mariko Kawamura, Satoko Asahi, Yuji Tameshige, Yoshikazu Maeda, Makoto Sasaki, Tomoyasu Kumano, Satoshi Kobayashi, Hiroyasu Tamamura, Toshifumi Gabata

**Affiliations:** 1 Proton Therapy Center, Fukui Prefectural Hospital, Fukui 910-8526, Japan; shigerad@staff.kanazawa-u.ac.jp (S.T.); k-yamamoto-7m@Pref.fukui.lg.jp (K.Y.); myuntaroh@yahoo.co.jp (M.M.); sayu.b4242@gmail.com (S.B.); y-satou-xn@pref.fukui.lg.jp (Y.S.); y-tameshige-af@pref.fukui.lg.jp (Y.T.); y-maeda-ce@pref.fukui.lg.jp (Y.M.); m-sasaki-hl@pref.fukui.lg.jp (M.S.); h-tamamura-8e@pref.fukui.lg.jp (H.T.); 2 Department of Radiotherapy, Kanazawa University Hospital, Kanazawa, Ishikawa 920-8641, Japan; t.kumano@staff.kanazawa-u.ac.jp; 3 Department of Radiology, Nagoya University Hospital, Nagoya, Aichi 466-8560, Japan; mkawamura@med.nagoya-u.ac.jp; 4 Department of Radiology, University of Fukui Hospital, Eiheiji, Fukui 910-1193, Japan; asahis@u-fukui.ac.jp; 5 Department of Radiology, Kanazawa University Graduate School of Medical Sciences, Kanazawa, Ishikawa 920-8641, Japan; satoshik@staff.kanazawa-u.ac.jp (S.K.); tgabata@icloud.com (T.G.)

**Keywords:** hepatocellular carcinoma, 4-dimensional CT planning, respiratory-gated irradiation, proton beam therapy

## Abstract

We evaluated the effectiveness and toxicity of proton beam therapy (PBT) for hepatocellular carcinomas (HCC) >5 cm without fiducial markers using four-dimensional CT (4D-CT) planning. The subjects were 29 patients treated at our hospital between March 2011 and March 2015. The median total dose was 76 Cobalt Gray Equivalents (CGE) in 20 fractions (range; 66–80.5 CGE in 10–32 fractions). Therapy was delivered with end-expiratory phase gating. An internal target volume (ITV) margin was added through the analysis of respiratory movement with 4D-CT. Patient age ranged from 38 to 87 years (median, 71 years). Twenty-four patients were Child–Pugh class A and five patients were class B. Tumor size ranged from 5.0 to 13.9 cm (median, 6.9 cm). The follow-up period ranged from 2 to 72 months (median; 27 months). All patients completed PBT according to the treatment protocol without grade 4 (CTCAE v4.03 (draft v5.0)) or higher adverse effects. The two-year local tumor control (LTC), progression-free survival (PFS), and overall survival (OS) rates were 95%, 22%, and 61%, respectively. The LTC was not inferior to that of previous reports using fiducial markers. Respiratory-gated PBT with 4D-CT planning without fiducial markers is a less invasive and equally effective treatment for large HCCs as PBT with fiducial markers.

## 1. Introduction

Hepatocellular carcinoma (HCC) is a common cancer in East Asia, including Japan, where hepatitis B and hepatitis C infection are prevalent [1]. In HCC treatment, resection, liver transplantation, and radiofrequency ablation (RFA) are designated as curative treatments and transcatheter arterial chemoembolization (TACE) and sorafenib chemotherapy are designated as palliative treatments in the Barcelona Clinic Liver Cancer (BCLC) staging system [2]. Resection appears to be the most effective treatment for HCC; however, it is invasive and poorly tolerated by sicker patients [3]. RFA is less invasive than resection. Its overall survival rate appears to be equivalent to that of surgical resection on small HCCs [4]; however, it is difficult to treat tumors >3 cm, or close to the hepatic artery, portal vein, and/or intestines [5].

Classic photon radiation therapy has rarely been used in HCC because the dose tolerance of normal liver tissue is considerably lower than that necessary for tumor control. Recently, highly conformal radiotherapy, such as stereotactic body radiation therapy (SBRT), has been reported to achieve good control for small HCCs with a tolerable liver dose [6,7]. However, for larger HCCs, photon therapy cannot easily provide adequate coverage of the target without increasing the risk of radiation-induced liver disease [8]. 

The excellent dose distribution in proton beam therapy (PBT) makes it possible to treat large liver tumors without a high dose to the normal liver. Recent studies have reported that PBT for HCC achieves good local control with less toxicity than photon therapy [9,10]. 

The liver moves in association with respiration [11,12,13]. There are many techniques to reduce the internal target volume (ITV) margin by decreasing respiratory motion using oxygen inhalation, abdominal compression, respiratory-gated treatment with/without implanting fiducial markers, and a voluntary breath-holding device [9,10,14,15,16]. In SBRT and PBT for liver neoplasms, fiducial markers are often used to aid in positioning [9,10,14]. Although complications are rare, implanting fiducial markers in the liver is an invasive procedure [14,17,18]. Using four-dimensional computed tomography (4D-CT) planning with fiducial markers has been reported to be highly accurate [19,20]. Combining the 4D-CT planning technique and adaptive PBT with frequent evaluation of the target during treatment may allow for PBT without fiducial markers. We evaluated the effectiveness and toxicity of 4D-CT planning for PBT for large HCCs (>5 cm) without fiducial markers.

## 2. Results

### 2.1. Toxicities

All patients completed PBT. Twenty-nine patients were followed up until death or until July 2017. Median follow-up time was 27 months (range, 2–72 months) (Table 1).

Acute toxicity occurred in one patient, characterized by Grade 3 hyperbilirubinemia during treatment. Other patients had skin reactions ≤ Grade 2. Six patients experienced late toxicity; two of Grade 3 pleural effusion and one of Grade 3 ascites, and one patient each with a Grade 2 rib fracture, radiation pneumonitis, and erosions of the ascending colon. No patient had late treatment-related toxicity > Grade 3.

### 2.2. Survival

**Figure 1:**
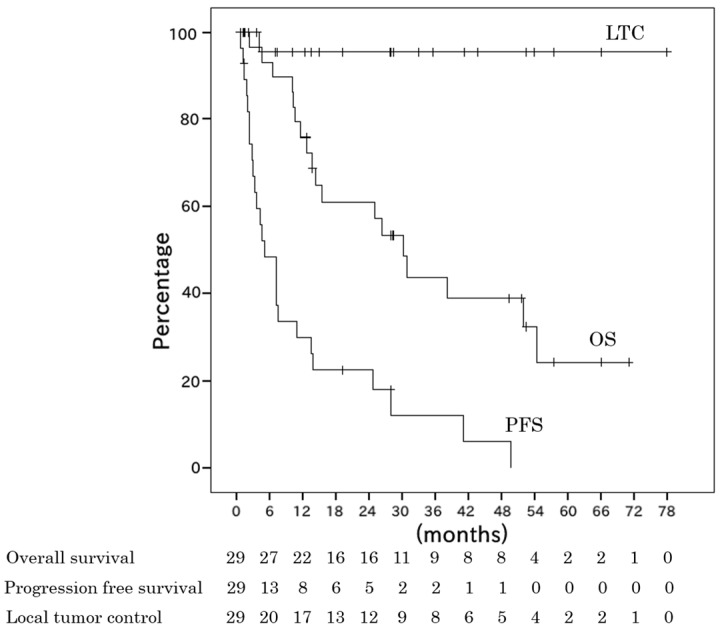
Kaplan-Meier estimates of overall survival (OS), progression-free survival (PFS) rates, and local tumor control (LTC) for 29 patients. The median overall survival period was 26.9 months (range, 2.4–72.3 months). The median progression-free survival period was 4.7 months (range, 0.7–50.6 months). The two-year OS, PFS, and LTC rates were 61%, 22%, and 95%, respectively.

**Figure 2:**
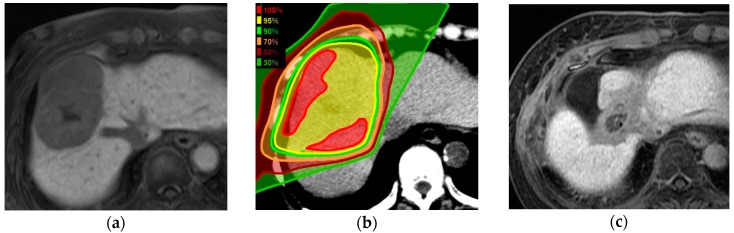
HCC (7.9 × 5.6 cm) treated by PBT (66 CGE/10 fractions) in a female in her eighties with hepatitis C liver cirrhosis, Child–Pugh class A. Tumor shows hypo-intensity in the hepatobiliary phase of gadolinium ethoxybenzyl diethylenetriamine pentaacetic acid-enhanced MRI (EOB-MRI) before treatment (**a**). The isodose lines displayed on planning CT (**b**). Three years on from PBT, the tumor was reduced in size on EOB-MRI. The normal liver in the irradiated area was atrophied, but severe liver damage was not observed (**c**).

The overall survival (OS) rates at two and four years after PBT were 61% (95% confidence interval (CI), 52–70%) and 39% (28–48%), respectively. The local tumor control (LTC) rates at two and four years were both 95% (95% CI, 91–100%). The progression-free survival (PFS) rates at one and two years were 30% (95% CI, 21–38%) and 22% (14–30%), respectively. The median PFS was five months (range, 1–51 months) (Figure 1). A case of successfully-treated HCC is shown in Figure 2. The patient had no severe complications over three years of follow-up.

There were no significant factors associated with OS in univariate and multivariate analyses. There were some significant factors associated with PFS in univariate analysis: T stage, size of tumor, planning target volume (PTV), operability, and history of prior treatment. In multivariate analysis, T stage and tumor size were significant factors associated with PFS (Table 2).

## 3. Discussion

Particle beam therapy is more focused than photon therapy because of the energy being concentrated in the Bragg peak. This property allows for highly conformal therapy for HCC with a much smaller dose to organs at risk (OARs) in comparison with photon therapy [21]. Many reports have been published about local control in PBT for HCC [9,10,22,23]. In our study, at two years, the OS was 61% and the local tumor control (LTC) was 95%. These are comparable with the results of previous reports (Table 3). However, our PFS rates at one and two years of 30% (95% CI, 21–38%) and 22% (14–30%) were lower than those of the previous studies, possibly because 17 of 29 cases had an advanced T-stage and multiple lesions (some treated with RFA or TACE). PBT for HCC, including our study, can achieve good local tumor control, but OS is unsatisfactory. Tumor size, tumor number, vascular invasion, and liver function are known prognostic factors in resected HCC patients [5], and are also prognostic in patients treated with PBT. We consider that early detection and prevention of cirrhosis of the liver caused from hepatitis virus, alcohol, and non-alcoholic steatohepatitis might improve the OS in PBT for HCC.

In statistical analysis, we could not find factors significantly influencing OS. However, the T stage, size of tumor, PTV, operability, and history of prior treatment were associated with PFS using univariate analysis. In multivariate analyses, T stage and tumor size were associated with PFS. Komatsu et al. reported that Child–Pugh classification was an independent risk factor for local recurrence in multivariate analysis and the age, performance status, and Child–Pugh classification significantly influenced OS in univariate analysis [24]. Tumor size, number of tumors, and vascular invasion were reported to be associated with PFS in resected HCC patients [25,26,27]. Our results are consistent with the PFS rates of patients with the risk factors described in the previous reports [9,25,26,27].

Reactions were generally mild and all patients completed PBT according to the treatment protocols with acceptable complications. Of 29 patients, one had acute Grade 3 hyperbilirubinemia. Six patients had late Grade 3 adverse events. No patient had Grade 3 liver dysfunction, bleeding, or inflammation of the digestive tract. Acute or late complications ≥ Grade 4 were not observed. Our results are consistent with the previous PBT reports using fiducial markers [9,10,22,23,24,28]. This suggests that our method offers effective treatment without the need for fiducial markers.

In PBT for HCC, a respiratory gating system with fiducial markers or the localization of lipiodol are two methods used for accurate positioning in the tumor after TACE [9,10,29]. Fiducial marker implantation in the liver is safe; however, some cases of bleeding and other complications have been reported [14,17,18,30]. In unfavorable cases, such as patients with poor liver function and coagulation abnormalities, fiducial marker implantation poses a risk. Accordingly, we used the diaphragm under fluoroscopic guidance as the landmark for liver tumors with a respiratory-gated system. Balter et al. [31] and Yang et al. [32] reported the diaphragm to be an acceptable anatomic landmark for liver motion. For precise treatment, we evaluated the target motion with 4D-CT planning and frequently re-examined the target with CT and/or MRI during the treatment period for adaptive PBT. We applied adaptive PBT using a respiratory-gated system with narrow gating at 17–25% of the duty cycle at end-expiration (about 1 s). Narrow gating can prolong the treatment time in patients with irregular respiration. To shorten treatment time, our patients had breath training to reduce irregular patterns [33]. Our method is useful for precise irradiation for treatment of large HCCs without inserting fiducial markers. 

There are some limitations to this study. The number of patients was limited, with retrospective analysis and a short follow-up period. Prospective studies with larger numbers of patients are needed to confirm the effectiveness and safety of 4D-CT planning PBT without fiducial markers for large HCC. Some guidelines do not accept radiation therapy and proton beam therapy for HCC as a standard treatment modality. Some guidelines suggested radiation therapy, or particle beam therapy for unresectable HCC, which would be difficult to treat with RFA. However, these guidelines do not recommend it [5]. Further evaluation in clinical studies is necessary.

Large tumor size is a negative prognostic factor as it is associated with resistance to radiation therapy. This is largely because of the hypoxic tumor microenvironment, and the increased expression of Poly(ADP-ribose) polymerase (PARP) and Hypoxia Inducible Factor 1a (HIF-1a) [34]. In our study, although good LTC was achieved, OS and PFS were not improved. Better adjuvant therapy may improve the treatment outcome of PBT for large HCCs. 

## 4. Patients and Methods

This retrospective study was approved by the research ethics committee of our hospital (IRB number 13–25) and written informed consent for this study was waived owing to its retrospective nature. Between March 2011 and March 2015, 118 patients were treated with PBT for HCC in our hospital. A total of 54 out of 118 patients had HCCs >5 cm in diameter; 29 of 54 patients who could be observed over 12 months or died within 12 months after PBT were enrolled. The 29 patients comprised 22 men and seven women, whose median age was 71 years (range, 38–87 years) (Table 1). HCCs were pathologically confirmed in four cases and clinically diagnosed in 25 cases based on the characteristic findings on dynamic CT and/or MRI, and serum level elevations of AFP and Des-gamma-carboxy prothrombin (PIVKA II). Treatment policy was discussed with surgeons and other physicians about the course of treatment at the tumor board in our hospital.

### 4.1. Proton Beam Therapy Planning

The patient setup and planning imaging have been previously reported [35]. We used respiratory-synchronized 4D-CT (Aquilion LB TSX-201A: Toshiba Medical Systems Co., Tochigi, Japan) for planning. Respiratory gating was controlled by monitoring abdominal wall motion with the laser sensor of a respiratory gating system (AZ-733V: Anzai Medical Co., Tokyo, Japan) under stable breathing (period of breathing 10–15 times/min by inducing rhythm using a metronome) [36]. CT data were reconstructed at a section thickness of 2 mm, and section interval (gap) of 0.4 mm. The field of view (FOV) was adjusted to match the physique of the patient. 

Targets were contoured at the end-expiratory phase using 4D-CT. Gross tumor volume (GTV) was delineated manually with contrast-enhanced CT and MRI. Clinical target volume (CTV) encompassed the GTV with a 0.5 cm margin in all directions. ITV was determined as CTV plus an additional margin due to respiratory movement calculated by the 4D-CT analysis. The internal margin due to respiratory movement was customized based on the amount of tumor motion visualized in the gating window at 17–25% duty cycle around the end of exhalation [37]. Planning target volume (PTV) encompassed the ITV with a 0.5 cm margin in all directions (Radiation treatment planning system: XiO-N; Elekta, Mitsubishi Electric Corporation, algorithm: proton pencil beam algorithm). We used two or more beam ports for PBT of liver tumors. Beam directions were selected to minimize dose to normal liver and to avoid the GI tract using patient collimators [28,38]. The total dose at the isocenter was prescribed to cover 95% of the PTV. 

### 4.2. Proton Beam Treatment

The PBT system (MELTHEA, Mitsubishi Electric Corporation, Kobe, Japan) uses proton beams ranging from 150 to 230 MeV, generated through a linear accelerator and synchrotron, spread out and shaped with ridge filters, a scatterer, a pair of wobbling magnets, multileaf collimators, and custom-made patient collimators and boluses, to conform the beams to the treatment planning data. The proton beams were controlled by the respiratory gating system, as simulated in treatment planning. It is an image-guided radiation therapy (IGRT) system with biplane kVp X-rays placed in the anterior–posterior and right–left directions using a robotic treatment table with six-axis correction of movement. It can correct setup errors by matching to the vertebral bodies and can show real-time movement of the diaphragm by kVp X-ray fluoroscopy. It can measure the distance between the diaphragm position at planning and the diaphragm position in peak-expiratory phase for gating. In our treatment, we adjusted the target position using vertebral bodies as landmarks, then adjusted for the displacement of the diaphragm by moving the treatment couch in the CC direction.

Seven protocols for respiratory-gated PBT (66.0–80.5 CGE in 10–38 fractions using 150, 190, or 230-MeV proton beams) were used in this study (Table 4), using an irradiation schedule of 5 fractions per week. The PBT protocol was selected depending on tumor location based on the previously reported studies [24]. A total dose of 66.0 CGE in 10 fractions was selected for tumors that were not adjacent to the GI tract or the porta hepatis. A total dose of 76.0 CGE in 20 fractions was selected for tumors adjacent to the porta hepatis. For tumors that were adjacent to the GI tract, a total dose of 76.0 CGE in 38 fractions or 70.4 CGE in 32 fractions was selected. Other protocols were employed as needed to minimize the OAR dose or accommodate the physical condition of the patient. 

The radiation dose was prescribed in CGE using a relative biological effectiveness value of 1.1, based on our preclinical experiments. Proton treatment beams were controlled for respiratory-gated delivery at 17–25% duty cycle around end-expiration (about 1 s) [37,39,40].

We evaluated the irradiated area by autoactivation imaging using positron-emission tomography/computed tomography (PET/CT) on the first day of PBT [41]. Autoactivation PET is suboptimal for evaluating irradiated dose or position in detail, thus we used this image for rough irradiated position confirmation. Therefore, we intended to evaluate the shape of the tumor, liver, and GI tract using CT images (slice thickness 3.75 mm) under breath-holding position at end of exhalation. We examined the target with CT and MRI at the time after 15–20 fractions (3–4 weeks after the beginning of treatment) for change of treatment planning [42]. The changes in the size of GTV, shape of liver, and GI tract were evaluated by rigid image registration using commercially available software (MIM Maestro: MIM Vista Corp, Cleveland, OH, USA). If GTV had shifted out of the PTV or OARs had moved into the PTV, we generated a new treatment plan. 

### 4.3. Follow-Up and Toxicity Evaluation

Follow-up evaluations were performed every three months after completion of PBT for the first three years, every six months in the following 3–5 years, and annually thereafter. At the follow-up visits, all patients received clinical and radiological examinations (abdominal CT and MRI). Toxicity was graded according to Common Terminology Criteria for Adverse Events, Version 4.03 (CTCAE v4.03 (draft v5.0)) (National Cancer Institute, Bethesda, MD, USA).

### 4.4. Statistical Methods

The Kaplan–Meier method was used for calculation of OS, PFS, and LTC. Multivariate analysis was performed using a Cox regression analysis. Variables using multivariate analysis were clinical factors (gender, age, chronic liver disease with viral infection, alcoholic liver, Child–Pugh class, operability, performance status, T classification, prior treatment, single mass or not [43], and whole liver volume), and planning factors (dose per fraction, tumor size, PTV). Hazard ratios (HRs) with 95% confidence intervals (CI) were calculated for each independent factor. The Kaplan–Meier method and the Cox regression analysis were performed using commercial (SPSS 20.0, IBM Corp., Armonk, NY, USA) software. A *p* value < 0.05 was defined as statistically significant.

## 5. Conclusions

Four-dimensional CT planning for respiratory-gated PBT without fiducial markers has the potential to be an effective and less invasive treatment method for large HCCs.

## Figures and Tables

**Table 1:** Characteristics of the patients and the tumors.

Characteristics	n
Number of patients	29
Gender, male/female	22/7
Age (years); median (range)	71 (38–87)
PS 0/1/2	21/7/1
Tumor size; median (range)	69 (50–139)
50–100 mm/>100 mm	22/7
CH HCV/HBV/alcoholic/others	5/11/4/9
Child Pugh A/B	24/5
Tumor thrombus PV/HV/bile duct	9/4/1
Prior treatment TACE/RFA/surgery	13/4/3
Operable/inoperable	7/22
Solitary/multiple (two or more)	14/15
Single nodular type/non single nodular type	5/24
T stage 1/2/3a/3b/4	4/8/9/7/1
GTV (cm^3^); median (range)	107 (23–1056)
PTV (cm^3^); median (range)	293 (138–1566)
Liver volume (cm^3^); median (range)	1310 (810–2259)

Abbreviations; PS: performance status; CH: chronic hepatitis; HCV: hepatitis C virus; HBV: hepatitis B virus; PV: portal vein; HV: hepatic vein; TACE: Transcatheter arterial chemoembolization; RFA: radiofrequency ablation; GTV: Gross tumor volume; PTV: planning target volume.

**Table 2:** Multivariate analysis of potential predictive factors for progression-free survival (PFS).

Variables	HR	95% CI	*p*-Value
T stage (T1-2/T3-4)	0.28	0.09–0.87	0.03
Tumor size (≤100 mm/>100 mm)	0.24	0.06–1.00	0.049
Volume of PTV (≤300 mL/>300 mL)	1.04	0.27–4.02	0.95
Operable/inoperable	0.63	0.14–2.74	0.54
History of previous treatment	0.95	0.33–2.77	0.92

Abbreviations; HR: hazard ratio; CI confidence interval; PTV: planning target volume.

**Table 3:** Reports of proton beam therapy for HCC.

Author	Number	Median Tumor Size (Range)	Median Treatment Dose	OS 2 Years	LTC 2 Years
Mizumoto et al. [9]	266	34 mm (6–130 mm)	72.6 CGE/22 Fr	61% (3 years)	87% (3 years)
Fukumitsu et al. [10]	51	28 mm (8–93 mm)	66.0 CGE/10 Fr	49% (3 years)	95% (3 years)
Sugahara et al. [22]	22	110 mm (100–140 mm)	72.6 CGE/22 Fr	36%	87%
Kimura et al. [23]	24	90 mm (50–180 mm)	72.6 CGE/22 Fr	52%	87%
This study	29	69 mm (50–139 mm)	76.0 CGE/20 Fr	61%	95%

Abbreviations; HCC: hepatocellular carcinoma; OS: overall survival; LTC: local tumor control; CGE: cobalt gray equivalent; Fr: fractions.

**Table 4:** Treatment protocols.

Total Dose (CGE)	Number of Fractions	Cases (*n* = 29)	The Number of Re-Plan	Fraction at Re-Plan (Cases)
66	10	4	-	-
76	20	13	1	13 (1)
80.5	23	1	-	-
80	25	1	-	-
67.5	25	1	-	-
70.4	32	5	5	20 (1), 22 (4)
76	38	4	4	20 (2), 30 (2)

Abbreviations; CGE: cobalt Gray equivalent.
