# Erratum: Shibata S.; et al. Proton Beam Therapy without Fiducial Markers Using Four-Dimensional CT Planning for Large Hepatocellular Carcinomas. *Cancers* 2018, *10*, 71

**DOI:** 10.3390/cancers10120508

**Published:** 2018-12-11

**Authors:** Satoshi Shibata, Shigeyuki Takamatsu, Kazutaka Yamamoto, Miu Mizuhata, Sayuri Bou, Yoshitaka Sato, Mariko Kawamura, Satoko Asahi, Yuji Tameshige, Yoshikazu Maeda, Makoto Sasaki, Tomoyasu Kumano, Satoshi Kobayashi, Hiroyasu Tamamura, Toshifumi Gabata

**Affiliations:** 1 Proton Therapy Center, Fukui Prefectural Hospital, Fukui 910-8526, Japan; shigerad@staff.kanazawa-u.ac.jp (S.T.); k-yamamoto-7m@Pref.fukui.lg.jp (K.Y.); myuntaroh@yahoo.co.jp (M.M.); sayu.b4242@gmail.com (S.B.); y-satou-xn@pref.fukui.lg.jp (Y.S.); y-tameshige-af@pref.fukui.lg.jp (Y.T.); y-maeda-ce@pref.fukui.lg.jp (Y.M.); m-sasaki-hl@pref.fukui.lg.jp (M.S.); h-tamamura-8e@pref.fukui.lg.jp (H.T.); 2 Department of Radiotherapy, Kanazawa University Hospital, Kanazawa, Ishikawa 920-8641, Japan; t.kumano@staff.kanazawa-u.ac.jp; 3 Department of Radiology, Nagoya University Hospital, Nagoya, Aichi 466-8560, Japan; mkawamura@med.nagoya-u.ac.jp; 4 Department of Radiology, University of Fukui Hospital, Eiheiji, Fukui 910-1193, Japan; asahis@u-fukui.ac.jp; 5 Department of Radiology, Kanazawa University Graduate School of Medical Sciences, Kanazawa, Ishikawa 920-8641, Japan; satoshik@staff.kanazawa-u.ac.jp (S.K.); tgabata@icloud.com (T.G.)

The authors wish to make the following corrections to this paper [1]:

The first author, Satoshi Shibata, was affiliated with Affiliation 1, “Proton Therapy Center, Fukui Prefectural Hospital, Fukui 910-8526, Japan”, in reference [1]. The authors would like to add Affiliation 2 to Satoshi Shibata, “Department of Radiotherapy, Kanazawa University Hospital, Kanazawa, Ishikawa 920-8641, Japan”. 

In reference 22 of the paper [1], the author name “Sugihara, S” should be changed to “Sugahara, S.”

These changes do not affect the scientific results. The manuscript will be updated and the original version will remain online on the article webpage, with a reference to this erratum. The authors would like to apologize for any inconvenience caused to the readers by this change.
